# Amoeboid migration in health and disease: Immune responses *versus* cancer dissemination

**DOI:** 10.3389/fcell.2022.1091801

**Published:** 2023-01-05

**Authors:** Samantha George, Joshua Alexander James Martin, Vittoria Graziani, Victoria Sanz-Moreno

**Affiliations:** Barts Cancer Institute, Queen Mary University of London, London, EC1M 6BQ, United Kingdom

**Keywords:** amoeboid migration, cancer, leukocyte, metastasis, migrastatics

## Abstract

Cell migration is crucial for efficient immune responses and is aberrantly used by cancer cells during metastatic dissemination. Amoeboid migrating cells use myosin II-powered blebs to propel themselves, and change morphology and direction. Immune cells use amoeboid strategies to respond rapidly to infection or tissue damage, which require quick passage through several barriers, including blood, lymph and interstitial tissues, with complex and varied environments. Amoeboid migration is also used by metastatic cancer cells to aid their migration, dissemination and survival, whereby key mechanisms are hijacked from professionally motile immune cells. We explore important parallels observed between amoeboid immune and cancer cells. We also consider key distinctions that separate the lifespan, state and fate of these cell types as they migrate and/or fulfil their function. Finally, we reflect on unexplored areas of research that would enhance our understanding of how tumour cells use immune cell strategies during metastasis, and how to target these processes.

## Introduction

Immune cell populations are our body’s housekeepers and defence mechanisms. They must respond and relocate to distant sites and are adapted to cross diverse environments. They are professionally motile, and their localisation and timely responsiveness are critical to perform their functions effectively (Chaplin, 2010). Motility is therefore key for immune cell development, maintaining tissue homeostasis, immunosurveillance, responding to injury/infection and eliminating pathogens (Chaplin, 2010). While mesenchymal migration requires moderate levels of Rho-ROCK to contract the cell rear and retract protrusions, fast amoeboid migration relies on hyper-activation of Rho-ROCK-driven actomyosin contractility (Friedl and Wolf, 2009). Most leukocytes adopt amoeboid migration, while some cancer cells also use this mode to aid in metastatic spread (Madsen and Sahai, 2010). Amoeboid migration is conserved across different species, detected in early eukaryotes, such as the *Dictyostelium* genus (Barry and Bretscher, 2010) and across mammalian cells (Titus and Goodson, 2017). It is also observed during embryonic development (Richardson and Lehmann, 2010; Ruprecht et al., 2015), in primordial germ cells (Fujimoto et al., 1977) and in adult tissues, including neurons (Amini et al., 2022), satellite muscle stem cells (Otto et al., 2011), leukocytes and malignant cells (Lämmermann and Germain, 2014). Importantly, under physical confinement, both cancer and immune cells exhibit the fastest amoeboid migration described so far (Liu et al., 2015). We highlight up-to-date comparisons between amoeboid migration of immune and cancer cells, and suggest how cancer cell amoeboid migration could be targeted to prevent metastatic spread.

## Cytoskeletal dynamics in cancer and immune amoeboid cells

### Rho-ROCK-myosin II cytoskeletal regulation

During an immune response, leukocytes remodel their cytoskeleton to allow rapid amoeboid migration. This behaviour is observed for neutrophils, T cells, B cells, monocytes, macrophages and dendritic cells (Guenther, 2022), although macrophages and dendritic cells can also adopt mesenchymal migration (Friedl and Weigelin, 2008). Visualisation of leukocyte amoeboid migration has been possible using 3D models and *in vivo* imaging (Nourshargh and Alon, 2014), with neutrophils showing the highest speeds up to 30 μm/min (Friedl and Weigelin, 2008). Rho-ROCK-driven myosin contractility drives amoeboid leukocyte migration, with short-lived pseudopods at the cell front and a uropod at the rear (Bros et al., 2019; Eddy et al, 2000). In two dimensions (2D), cell migration has been described as a cyclical process: generation of actin-rich membrane protrusions, substrate-receptor engagement, actomyosin contraction of the cell rear and subsequent forward motion (Friedl and Weigelin, 2008). While cell polarity is key for directionality, leukocytes are guided by chemotactic gradients (Amano et al., 2010). Cytoskeletal polarisation is largely driven by signals mediated by G-protein coupled receptors (GPCRs) and receptor tyrosine kinases (RTKs). PI3K-PIP-Akt downstream signalling promotes actin polymerisation and pseudopods, as well as downstream T cell receptor (TCR) signalling involving Rac and Cdc42 activation (Enomoto et al., 2005; Gambardella and Vermeren, 2013). However, under confinement, leukocyte migration is largely adhesion-independent, utilising retrograde actin flow and Rho-ROCK-driven actomyosin contractility (Kameritsch and Renkawitz, 2020). This enhanced contractility allows cells to squeeze through small gaps whilst maintaining a stable cortex and cellular integrity (Bendix et al., 2008), but is also crucial for rear detachment, retraction (Alblas et al., 2001; Lämmermann and Sixt, 2009), differential integrin expression (Liu et al., 2002). Amoeboid leukocytes can still generate force and forward motion without strong substrate engagement (Reversat et al., 2020), where confinement and low adhesion can induce amoeboid behaviour (Liu et al., 2015). Low adhesion also allows amoeboid cells to migrate faster through increased cortical actin tension (Lämmermann et al., 2008). Integrins play a role in pseudopod selection (Andrew and Insall, 2007) and cellular contractility promotes uropod de-adhesion and rear retraction during amoeboid migration in several immune types (Worthylake et al., 2001; Sánchez-Madrid and Serrador, 2009). Consequently, myosin inhibition abrogates neutrophil rear retraction (Eddy et al., 2000), whilst reducing Rho-ROCK activity induces an amoeboid-to-mesenchymal transition (AMT) within macrophages (Gui et al., 2014). Therefore, actomyosin contractility is a crucial driver of leukocyte amoeboid migration.

Tumour cells can acquire amoeboid characteristics, which has been reported in several cancer types, including melanoma, breast cancer, lymphoma, leukaemia, liver cancer, sarcoma and prostate cancer (Crosas-Molist et al., 2017; Pan et al., 2018; López-Luque et al., 2019; Graziani et al., 2022). This amoeboid behaviour can be induced by cytokines (Sanz-Moreno et al., 2011; Georgouli et al., 2019) and mechanical cues (Liu et al., 2015; Khoo et al., 2019). Amoeboid cells harbour elevated levels of Rho-ROCK signalling, which support migration, invasion and metastasis (Crosas-Molist et al., 2021). Similarly to immune cells, enhanced contractility in cancer cells couples movement of the cell rear to the front, allowing cell body translocation, squeezing through confined environments and maintenance of cellular integrity (Sahai and Marshall, 2003; Wyckoff et al., 2004; Wyckoff et al., 2006; Liu et al., 2015). Both immune and tumour amoeboid cells use blebs as functional membrane protrusions (Paluch and Raz, 2013). Blebs are short-lived, formed due to increased hydrostatic pressure and can be induced by confinement (Ibo et al., 2016) and/or low-adhesion, which rely less on long-lasting substrate interactions (Schick and Raz, 2022). Amoeboid migration modes include ‘pseudopodial’ blebs governed by dynamic actin assembly in leukocytes (Lämmermann and Sixt, 2009), alongside leader or ‘stable’ blebs, which remain un-retracted during migration (Schick and Raz, 2022). However, less is known about how polarised bleb formation can drive directional migration. In summary, Rho-ROCK is a key driver of bleb-based amoeboid migration, enabling fast movement for both leukocytes and cancer cells.

### Amoeboid migration and cellular plasticity

Amoeboid leukocytes display context-dependent migration strategies, guided by soluble (chemotaxis) or immobilised (haptotaxis) chemokines, in addition to haptokinesis (2D migration during vascular crawling), durotaxis (rigidity gradients) and tenertaxis (path of least resistance) (Schimmel et al., 2017). Cancer cells also adopt diverse migratory strategies, including collective or individual migration (De Pascalis and Etienne-Manneville, 2017), similarly guided by chemotaxis (Roussos et al., 2011), haptotaxis and haptokinesis (Lu et al., 2014; Oudin et al., 2016). However, it is unclear whether this is specific to amoeboid migration. Plasticity between individual mesenchymal and amoeboid migration has been observed and the mesenchymal-to-amoeboid transition (MAT) can be considered part of the epithelial-to-mesenchymal transition (EMT) spectrum (Graziani et al., 2022).

Leukocytes have evolved to display migratory plasticity to cross diverse barriers (Laurent et al., 2017). However, tumour cells lack this pre-programmed advantage, but instead hijack this migratory plasticity *via* transcriptional rewiring. Induction of cellular plasticity arises from aberrant mutations, involving the adaptability of migration strategies (Friedl and Wolf, 2009; Te Boekhorst et al., 2016). Mechanical constraints can trigger plasticity, where macrophage and cancer cell migration is influenced by matrix organisation (Van Goethem et al., 2010; Poltavets et al., 2018; Čermák et al., 2018). In tumours, the shift between elongated-mesenchymal and rounded-amoeboid migration modes are in part governed by the balance of Rho and Rac signalling (Sahai and Marshall, 2003; Sanz-Moreno et al., 2008), cytokine signalling (Sanz-Moreno et al., 2011; Cantelli et al., 2015; Georgouli et al., 2019) and mechanical sensing (Liu et al., 2015). Tumour cell mesenchymal migration has been associated with protease-dependence, while amoeboid migration can be protease-independent (Sahai and Marshall, 2003; Carragher et al., 2006; Sanz-Moreno et al., 2008) or protease-dependent (Orgaz et al., 2014). Leukocytes and tumour cells modulate this proteolytic dependency through the generation of actin-rich podosomes and invadosomes, respectively, which regulate points of ECM attachment and localised matrix metalloproteinase (MMP) release (Murphy and Courtneidge, 2011). Overall, both immune and tumour cells share parallels of cellular plasticity to overcome any barriers during dissemination.

## Shared strategies for effective dissemination

### Amoeboid-driven pro-inflammatory signalling

STAT3 (Yu et al., 2009; Kaplan, 2013) and NF-κB (Liu et al., 2017) act as central transcriptional hubs controlling inflammatory secretion in immune responses, and drive cancer progression and amoeboid dissemination (Pan et al., 2018; Taniguchi and Karin, 2018; Owen et al., 2019). Immune cells produce pro-inflammatory factors, including IFNγ and IL-1β, to activate immune responses (Lacy and Stow, 2011). While, regulatory T cells ensure timely immune response termination through secretion of immunomodulatory factors, namely IL-10 and TGF-β (Marrack et al., 2010; Kane et al., 2014). Furthermore, IL-6 regulates T cell recruitment through selective MAPK, PI3K and JAK/STAT activation (Weissenbach et al., 2004; Fielding et al., 2008). Comparatively, Rho-ROCK signalling sustains cancer amoeboid behaviour through cytokine secretion-driven positive feedback *via* IL-6 family cytokines/GP130-JAK1-STAT3 (Sanz-Moreno et al., 2011), TGF-β-SMAD2-CITED1 (Cantelli et al., 2015) and IL-1α-NF-κB (Georgouli et al., 2019). Therefore, a key difference is that immune responses are halted to resume homeostasis (Ruland, 2011), whereas this mechanism is lost in chronically inflamed cancers, which could be exploited for therapeutic interventions.

Rho-ROCK regulates secretion of pro-inflammatory factors, IL-1α and IL-8, and immunosuppressive factors, TGF-β and IL-10. This amoeboid cancer cell secretome supports endothelium attachment and vascular permeability, alongside the induction of tumour-promoting macrophages (Cantelli et al., 2015; Georgouli et al., 2019). Moreover, tumour and immune cells utilise cytokine and chemokine signalling to regulate their microenvironment and invasiveness (Sokol and Luster, 2015). However, evidence linking cytokine signalling to immune cell amoeboid migration and ROCK signalling is missing. Whether chemokines regulate Rho-ROCK actomyosin in amoeboid cancer cells is also not fully understood. Therefore, a better understanding of these pathways could present another avenue for targeting amoeboid cancer cells. Delineating cancer-specific pro-inflammatory signalling and non-canonical roles of these central transcription nodes will be crucial in tackling cancer-specific programmes (Baud and Karin, 2009; Siveen et al., 2014; Yu et al., 2020).

### Interstitial and transendothelial migration

During interstitial migration, leukocytes use tenertaxis to migrate towards preferential crossing points to minimise the tissue remodelling required (Muller, 2013). Interstitial migration is further directed by chemotactic gradients, including CXCL8-CXCR2, CXCL2-CXCR2, and CCL21-CCR7 chemokine signalling (Lam and Huttenlocher, 2013), whereas amoeboid cancer cell chemotaxis is reliant on CXCL12-CXCR4 (Wyse et al., 2017) and CCL25-CCR9 (Zhang et al., 2011), while CCL2-CCR2 activates MEK-ROCK2-myosin II axis (Wong et al., 2020). CXCL12-CXCR4 drives RhoA-dependent bleb-based migration (Wyse et al., 2017) which promotes rapid amoeboid cancer cell interstitial migration, also driven independently of integrin β1 (Tozluoğlu et al., 2013). In leukocytes, integrins are not essential for interstitial migration; instead, cells become reliant on increased actin polymerisation and actomyosin contractility (Nourshargh et al., 2010). However, adhesions in amoeboid cells can be induced by inflammatory signals, such as TNFα, to aid in context-dependent stoppage and retention (Lokuta and Huttenlocher, 2005). Generally, amoeboid cancer cells are less reliant on adhesion (Carragher et al., 2006), while β1 integrins are required for their adhesion to collagen I (Pinner and Sahai, 2008b; Sanz-Moreno et al., 2008). Furthermore, leukocytes and tumour cells use podosomes (Linder and Aepfelbacher, 2003; Wiesner et al., 2014) and invadopodia, respectively (Linder and Kopp, 2005; Linder, 2009), for interstitial tissue migration and to cross the endothelium (Bravo-Cordero et al., 2012). Podosomes mediate ECM attachment and act as active hubs of MMP release (Murphy and Courtneidge, 2011), while invadopodia, which are typically longer-lived, also function in degrading matrix (Spuul et al., 2014). Amoeboid cancer migration is less reliant on proteolysis as enhanced contractility allows matrix deformation, but Rho-ROCK signalling in amoeboid cancer cells still supports MMP secretion and matrix degradation (Orgaz et al., 2014). Whether amoeboid cancer cells can form invadosomes in certain conditions is unknown, however it has been shown that RhoC-ROCK promotes ovarian carcinoma invasion through cortactin/cofilin-mediated invadosome formation and MMP-mediated degradation (Semprucci et al., 2016).

When moving between dense interstitial tissue and vasculature, cells use local signals to identify suitable intravasation and extravasation sites (Subramanian et al., 2020) and adapt their cytoskeleton (Mihlan et al., 2022). Rho-ROCK signalling promotes the transendothelial migration (TEM) process (Honing et al., 2004). Establishing interactions with endothelium is also key for cancer cells and leukocytes to cross the vasculature. Integrins, CAMs, selectins and N-cadherin on the surface of endothelial and transmigrating cells are key for this process (Ley et al., 2007). Leukocytes rely on integrins, such as α4β1, αLβ2, α4β7 and αMβ2 for firm adhesion and crawling along the endothelium before extravasation (Mitroulis et al., 2015). Whilst cancer cells use similar mechanisms to cross the endothelium, they are not as reliant on integrins (Miles et al., 2008). For example, leukocytes use PSGL-1, LFA-1, JAM-C, PECAM-1, and CD-99, whereas cancer cells rely on PSGL-1, MUC1, P-, L- and E-selectins (Strell and Entschladen, 2008; Muller, 2011). Furthermore, leukocytes maintain adhesion molecule expression alongside chemokine-driven “inside-out” signalling to activate integrins and promote adhesion to endothelium (Nourshargh and Alon, 2014). On the other hand, CXCR4 expression (Jin et al., 2012) and TGF-β-driven Rho-ROCK-myosin II (Lamouille et al., 2014; Cantelli et al., 2015) aid cancer cell-endothelial adhesion. Both cancer and immune cells then use these interactions to alter endothelial cell cytoskeleton to aid TEM (Schimmel et al., 2017; Rodenburg and van Buul, 2021). When leaving the vasculature, extravasation sites are selected by leukocytes (Nourshargh and Alon, 2014) and tumour cells (Sökeland and Schumacher, 2019) based on the production of chemotactic factors by inflamed tissue. Consequently, chemokine receptor expression also influences leukocyte tissue tropism (Olson and Ley, 2002) and organ-specific metastasis tropism (Marcuzzi et al., 2018). Therefore, targeting chemokine and/or Rho-ROCK signalling in amoeboid cancer cells could prevent cancer-endothelium interactions, TEM and metastatic dissemination to peripheral secondary sites. However, a better understanding of the amoeboid cancer cell adhesome will allow specific targeting of amoeboid cancer cell-endothelium interactions, whilst sparing leukocyte-endothelium interactions.

### Survival in circulation

In circulation, leukocytes rely on their plastic nature to survive *via* metabolic adaptation, integrin-dependent adhesions (Alon and van Buul, 2017) and extensive nuclear and cytoplasmic deformations to cope with shear stress (Majidpoor and Mortezaee, 2021; Perea Paizal et al., 2021). Comparatively, tumour cells struggle to survive in circulation, with an estimated half-life of only 1–2.4 hours (Meng et al., 2004) and <0.01% of circulating tumour cells successfully extravasate to secondary organs (Langley and Fidler, 2011). This represents a significant discrepancy to leukocytes, particularly monocytes that can survive in circulation for days (Patel et al., 2017). However, tumours cells that survive in circulation show increased actomyosin signalling (Moose et al., 2020) and upregulate pro-survival and proliferation signalling pathways, such as FAK, ERK, and Akt (Alanko et al., 2015; Douma et al., 2004). This is achieved through increased adhesion-dependent ‘shielding’ with platelets (Palumbo et al., 2005) and monocytes/macrophages (Gil-Bernabé, et al., 2012), to protect from shear stress and immune responses (Strilic and Offermanns, 2017). Targeting these pathways could hinder survival in circulation and perturb secondary site seeding and metastasis.

## Differences between amoeboid immune and cancer cells

We have explained how immune cells paradoxically share a panel of similarities with tumour cells. In the search for cancer amoeboid biomarkers, features and pathways that are unique to cancer cells and absent in anti-tumoural immune cells would be interesting. Genetic mutations, lifespan and differentiation status are unique characteristics of tumour cells.

### Mutational status

Normal cells harbour a lower mutation burden than cancers originating in the same organs, however certain immune cells, such as T cells and B cells, generate programmed somatic mutations to create the antigen repertoire necessary to exert their functions (Machado et al., 2022). Random mutations can also occur in immune cells and these can lead to lymphoid malignancies or other pathological conditions (Abplanalp et al., 2021). Tumours harbour mutation patterns caused by random errors occurring during DNA replication, either inherited or by environmental factors (Tomasetti et al., 2017). Frequently altered genes across tumour types contribute to tumour fitness (Bailey et al., 2018; Buisson et al., 2019) and, in certain tumours, lead to aberrant activation of Rho-ROCK-myosin II signalling, supporting a cancer amoeboid phenotype (Graziani et al., 2022).

BRAF and NRAS cooperate with Rho-ROCK (Qiu et al., 1995; Sahai et al., 1998) for transformation, and as such *BRAF-* and *NRAS*-mutant melanomas harbour amoeboid characteristics (Orgaz et al., 2020; Rodriguez-Hernandez et al., 2020). Inhibition of either BRAF^V600E^ or MEK results in loss of amoeboid behaviour by reducing myosin activity (Orgaz et al., 2020). BRAF inhibitors are available, yet resistance is a clinical challenge (Flaherty et al., 2012; Sanchez et al., 2018). BRAF-resistant melanomas adapt to therapy by altering cytoskeletal gene expression, which leads to myosin II activity restoration (Orgaz et al., 2020). Rho-ROCK-myosin II axis also cooperates with *MYC* oncogenes (Dyberg et al., 2017; Talamillo et al., 2017). *MYC*-dependent Rho-ROCK-myosin II activation sustains glioblastoma growth and invasion (Talamillo et al., 2017), while increased ROCK2 expression characterises high-risk neuroblastoma and correlates with poor patient survival (Dyberg et al., 2017). In acute myeloid leukaemia (AML), the oncogenic forms of *FLT3, BCR-ABL*, and *KIT* drive PI3K-Rho-ROCK-myosin II activation and *in vivo* tumour growth (Mali et al., 2011). In diffuse gastric cancer, *RhoA^Y42C^* gain-of-function mutations are characterised by a 12-fold increased capability to bind ROCK (Kakiuchi et al., 2014). Nonetheless, whether *MYC*, *RhoA* ^*Y42C*^, *FLT3*, *BCR-ABL,* and *KIT* oncogenes promote amoeboid behaviour is unknown.

Importantly, the cancer amoeboid cellular state cannot solely be explained by oncogenic signalling. Environmental factors such as hypoxia (Lehmann et al., 2017), production of arachidonic acid by cells under confinement (Lomakin et al., 2020) and low levels of reactive oxygen species (ROS) (Herraiz et al., 2016; Rodriguez-Hernandez et al., 2020) support amoeboid behaviour. Moreover, some cancer cells present constitutive amoeboid behaviour, suggesting an ‘amoeboid cellular memory’ regulated at the epigenetic level (Graziani et al., 2022).

### Lifespan and function

Most human cells are characterised by a finite replicative potential (Hahn et al., 1999) and immune cells present a limited lifespan (Nayar et al., 2015). Short-lived immune cells include neutrophil and monocytes, which have a half-life of 13–19 hours and lifespan of 1–7 days, respectively (Patel et al., 2021). Monocyte-derived macrophages have a short lifespan, while tissue macrophages survive for six weeks (Plowden et al., 2004). Memory T cells live for approximatively six months, whereas naive T cells can live for up to nine years (Borghans and Ribeiro, 2017). Conversely, cancer cells need to become immortal to form a neoplasm (Hahn et al., 1999). In most normal cells, each division results in telomeric DNA shortening, which eventually causes genomic instability, senescence and apoptosis (Hahn et al., 1999). Stem cells and cancers maintain stable telomere length due to telomerase reactivation or lengthening (Gunes and Rudolph, 2013). However, telomerase inhibitors have been unsuccessful in the clinic (Bar and Thum, 2017; Akincilar et al., 2021) and alternative strategies to halt tumour replicative immortality are needed. Interestingly, the actin cytoskeleton mechanically regulates telomeres in a length- and timescale-dependent manner (Jokhun et al., 2018), where cortical actomyosin-based contraction may influence replicative potential in amoeboid cells. However, whether ROCK plays a role in telomere lengthening is unknown. Amoeboid melanoma cells display cancer stem cell properties *in vitro* and *in vivo* (Rodriguez-Hernandez et al., 2020) and a pro-survival advantage during the acquisition of resistance to anti-melanoma therapies (Orgaz et al., 2020). Stemness is sustained by WNT11-FZD7-DAAM1 signalling and targeting this pathway could potentially inhibit self-renewal capabilities of melanoma amoeboid cells (Rodriguez-Hernandez et al., 2020).

### Differentiation status

Immune cells differentiate to perform specific functions (Maslova et al., 2020), while tumours vary in differentiation status (Vega et al., 2019). Undifferentiated tumour cells are often characteristic of advanced disease and poor prognosis (Jogi et al., 2012). Tumour de-differentiation has been linked with EMT (Wang and Unternaehrer, 2019) and cancer amoeboid cells could be considered part of the EMT spectrum (Graziani et al., 2022). Consistently, amoeboid cells support tumour-initiating abilities in melanoma, where ALDH1 is a strong biomarker of self-renewal (Rodriguez-Hernandez et al., 2020) and podoplanin^high^ amoeboid melanoma cells are linked to a clear de-differentiation state (de Winde et al., 2021). Therefore, differentiation therapies targeting amoeboid cancer stem cells could halt tumour progression and prevent relapse, but more work is needed in this field.

Undifferentiated tumours have been linked to immune suppression (Mo et al., 2016; Ahn et al., 2021; Yi et al., 2021). Checkpoint inhibitors have showed clinical activity in a variety of tumours, although resistance is still a challenge (Sharma et al., 2017). Immunotherapy (IT)-resistant melanomas harbour high actomyosin and amoeboid features, but combining IT with ROCK inhibitors improved responses (Orgaz et al., 2020). These effects are due in part to decreased immunosuppressive populations and unaffected CD4^+^CD8^+^ infiltration. Hence, there may be a therapeutic window in which ROCK inhibitors can be used without affecting anti-tumour immune responses while reducing pro-tumorigenic populations, such as macrophages and regulatory T cells (Orgaz et al., 2020). Strategies to eradicate amoeboid cells with stemness and immunosuppressive attributes should be considered for future therapeutic strategies while reliable ‘amoeboid biomarkers’ will be crucial.

### Amoeboid behaviour in haematological malignancies

The immune system is a double-edged sword in cancer as it can boost or hinder tumour development (Lakshmi Narendra et al., 2013). When genetic alterations occur in immune cells, haematological malignancies can develop (Abplanalp et al., 2021). While adherent cells require optimal levels of myosin II to survive (Schipper et al., 2019), immune cells and haematological cancer cells exhibit hard-wired capacities to live in environments without strong adhesive interactions (Suresh, 2007; Yamada and Sixt, 2019). While in solid tumours the activation of Rho-ROCK-myosin II is coupled to metastatic potential, in haematological malignancies it is also linked with enhanced proliferation and survival (Crosas-Molist et al., 2021). In AML, PI3K-Rho-ROCK signalling is highly upregulated, whereby ROCK inhibition impairs their proliferative capacity (Mali et al., 2011). Moreover, Rho-ROCK promotes metastasis of acute lymphoblastic leukaemia (ALL) cells in response to CCL25 (Zhang et al., 2011), while KIF13A regulates RhoB vesicular recycling promotes bleb-based amoeboid migration in ALL (Gong et al., 2018). Moreover, in chronic lymphocytic leukaemia (CLL) the pro-survival protein ABL1 co-localises with F-actin structures to promote amoeboid migration (Hutchinson et al., 2014). On the other hand, STAT3 supports amoeboid migration in diffuse large B-cell lymphoma (DLBCL), *via* ARHGEF2-RhoA signalling, whereby JAK inhibition reduces dissemination (Pan et al., 2018). In chronic myelogenous leukaemia (CML), p210^bcr/abl1^ activates RhoA leading to amoeboid migration, where inactivation of RhoA is able to reverse this process (Daubon et al., 2008). This body of data suggests that Rho signalling is a promising target in haematological malignancies and inhibitors of this pathway should be explored.

## Discussion and future directions

There are important parallels between amoeboid leukocyte and tumour cell migration, driven by both cytokine signalling and Rho-ROCK activation. The rapid nature of amoeboid migration often allows the spread of cancer before its detection. Finding unique vulnerabilities of cancer cells that will not affect immune function will be important. We have explored how Rho-ROCK signalling drives key strategies during both immune responses and cancer cell dissemination, while diverse oncogenic drivers converge to activate the Rho-ROCK-myosin II axis. Since myosin II is a tuneable switch, its contribution to tumourigenesis may be context-, tumour stage- and microenvironment-dependent (Wang et al., 2019). Thus, it is important to select patients that will benefit from Rho-ROCK-myosin II signalling inhibition.

**FIGURE 1:**
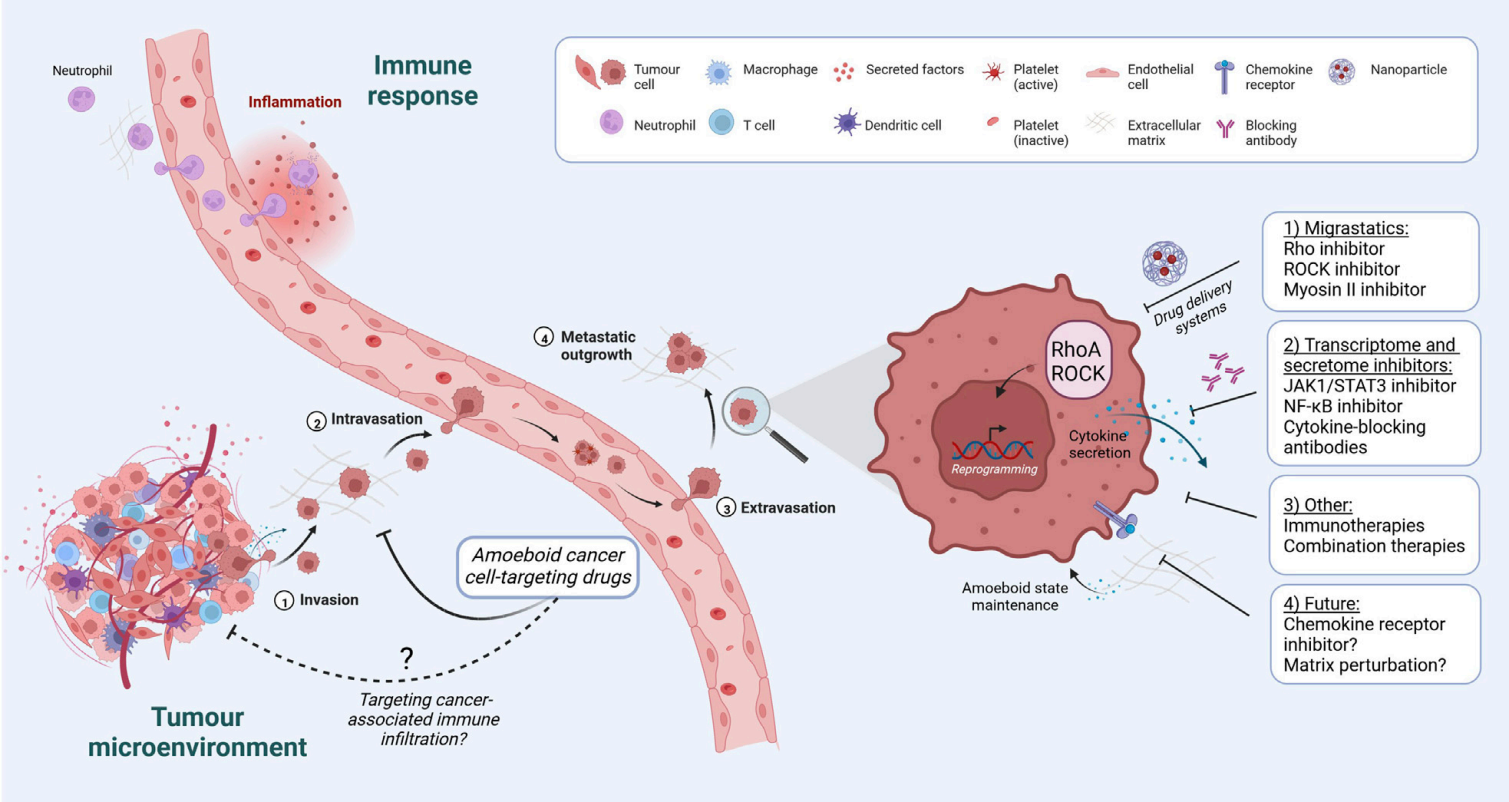
Targeting amoeboid cancer cells during tumour progression. Both leukocytes and tumour cells utilise amoeboid migration for their motility. We can aim to target distinct hallmarks of amoeboid cancer cells within the tumour microenvironment, whilst leaving homeostatic leukocytes intact. The effect of migrastatics on tumour-immune infiltrate is less understood, and should also be considered. Targeting approaches for amoeboid cancer cells are highlighted, from migrastatics, transcriptome and secretome inhibitors, immunotherapies and combination therapies, alongside future considerations for therapies such as chemokine receptor inhibitors.

ROCK inhibitors show clear anti-tumour effects in *in vivo* mouse models (Liu et al., 2009; Georgouli et al., 2019; Orgaz et al., 2020; Rodriguez-Hernandez et al., 2020; Kim et al., 2021). Targeting Rho GTPases within immune cells has been explored for the treatment of chronic inflammatory disorders (Biro et al., 2014). However, more work is needed to understand if ROCK inhibitors affect anti-tumour immunity (Kim et al., 2021). Migrastatics, including ROCK inhibitors, could be used to prevent metastatic disease (Gandalovičová et al., 2017; Maiques et al., 2021), but this needs to be carefully considered within the context of complex tumour environments and administration routes (Rath and Olson, 2012). ROCK inhibitors are approved to treat glaucoma (Honjo and Tanihara, 2018), cerebral vasospasm (Zhao et al., 2006) and graft vs. host disease (Ali and Ilyas, 2022), and are currently being tested for numerous diseases, including cancer (Crosas-Molist et al., 2021). ROCK inhibitors could be utilised for combination therapies, for example ROCK inhibitor/BRAF inhibitor and ROCK inhibitor/αPD-1 (Orgaz et al., 2020), alongside other chemotherapeutic agents (Kim et al., 2021), or *via* dual target inhibition (McLeod et al., 2020). Alternatively, local application for skin cancers, drug delivery systems (e.g., nanoparticles), antibody drug conjugates or oncolytic viruses could be explored, although these avenues are in early stage development (Senapati et al., 2018; Krishnan and Mitragotri, 2020; Yao et al., 2020). ‘Soft’ ROCK inhibitors have also been put forward in an effort to reduce systemic exposure and side effects (Boland et al., 2015). These options all contribute to the arsenal of treatment options (Figure 1; Table 1).

**TABLE 1:** Putative targets for amoeboid cancer cells.

Process	Amoeboid cancer targets	Putative anti-amoeboid cancer agents
Migration in confined environments	• Rho-ROCK-myosin II (Sahai and Marshall, 2003; Sanz-Moreno et al., 2008; Liu et al., 2015) and bleb formation (Paluch and Raz, 2013)	• Migrastatics and ROCK inhibitor (Gandalovičová et al., 2017; Maiques et al., 2021)
Interstitial migration	• ROCK-driven invasion *in vivo* (Wyckoff et al., 2006; Pinner and Sahai, 2008a)	• Migrastatics and ROCK inhibitor (Gandalovičová et al., 2017; Maiques et al., 2021)
	• Bleb-based migration *in vivo* (Tozluoğlu et al., 2013)	
	• CXCL12-CXCR4-driven bleb-based migration (Wyse et al., 2017)	• CXCR4i (Jin et al., 2012; Pernas et al., 2018)
Transendothelial migration	• Adhesion molecules: PSGL-1, integrin α4β1/β7 and MUC1 (Strell and Entschladen, 2008)	
	• TGF-β-RhoA driven endothelial adhesion (Cantelli et al., 2015)	• TGF-βi (Yap et al., 2020)
	• Aberrant CXCR4 expression (Jin et al., 2012)	• CXCR4i (Jin et al., 2012; Pernas et al., 2018)
Inflammation	• Rho-ROCK, STAT3, NF-κB, IL-6, IL-8, IL-10, TGF-β & IL-1α (Sanz-Moreno et al., 2011; Cantelli et al., 2015; Georgouli et al., 2019)	• JAK-STAT inhibitor (Qureshy et al., 2020)
		• TGF-βi (Yap et al., 2020)
		• IL6i (Mace et al., 2018)
		• STAT3i (Zou et al., 2020)
		• IL-1 and IL-1R1 blockade (Dinarello et al., 2012; Ruscitti et al., 2019)
		• IL8i (Bilusic et al., 2019)
Survival in vasculature	• Rho-ROCK driven pro-survival and proliferation pathways: FAK, ERK & Akt (Strilic and Offermanns, 2017; Kai et al., 2019; Moose et al., 2020)	• JAK-STAT inhibitor (Qureshy et al., 2020)
		• TGF-βi (Yap et al., 2020)
	• Tumour-platelet aggregates (Strilic and Offermanns, 2017; Palumbo et al., 2005)	• IL6i (Mace et al., 2018)
		• STAT3i (Zou et al., 2020)
		• IL-1 and IL1-R1 blockade (Dinarello et al., 2012; Ruscitti et al., 2019)
Mutational burden	• Oncogenes cooperating with Rho-ROCK-myosin II: *BRAF* and *NRAS* (Qiu et al., 1995; Sahai et al., 1998; Orgaz et al., 2020; Rodriguez-Hernandez et al., 2020); *MYC* (Talamillo et al., 2017); *FLT3*, *BCR-ABL*, *KIT* (Mali et al., 2011)	• ROCK inhibitor and combination ROCK inhibitor with targeted therapies (Orgaz et al., 2020)
Lifespan	• Self-renewal WNT11-FZD7-DAAM1 pathway (Rodriguez-Hernandez et al., 2020)	• WNT signalling cascade inhibitors (Harb et al., 2019)
Differentiation status and stemness	• Self-renewal WNT11-FZD7-DAAM1 pathway (Rodriguez-Hernandez et al., 2020)	• WNT signalling cascade inhibitors (Harb et al., 2019)
	• Immunosuppressive microenvironment (Mo et al., 2016; Ahn et al., 2021; Yi et al., 2021)
		• PD-1/PD-L1 monoclonal antibodies (Mo et al., 2016; Ahn et al., 2021; Yi et al., 2021)
		• Combination ROCK inhibitor and PD-1/PD-L1 monoclonal antibodies (Orgaz et al., 2020)

Alternative avenues could target amoeboid immunosuppressive secretion, including TGF-β (Cantelli et al., 2015; Giannelli et al., 2020; Yap et al., 2020). Beyond targeting individual amoeboid-dependent secreted factors, it could be possible to target related signalling nodes, such as the JAK-STAT and NF-κB pathways (Qureshy et al., 2020). Recently, combining STAT3 inhibitors and immunotherapy has shown encouraging results (Zou et al., 2020), while IL-1/IL-1R1 also represent potential targeting options (Dinarello et al., 2012; Ruscitti et al., 2019). Furthermore, immunotherapy could be combined with chemokine signalling inhibition, for example CXCR4 antagonist (Balixafortide) with Eribulin in metastatic breast cancers (Pernas et al., 2018), and CCR2 inhibition (CCX872) with αPD-1 treatment in pancreatic adenocarcinoma tumours (Jung et al., 2016). Therefore, these strategies could represent the most effective way to target amoeboid cancer-dependent secretion and chemotactic migration. However, a better understanding of chemokine signalling in amoeboid cancer cells is required to elucidate whether chemokine signalling inhibition would be effective. Finally, amoeboid cancer cells also retain cancer stem cell-like properties (Rodriguez-Hernandez et al., 2020). Developing targeted therapies to eradicate cancer stem cells is challenging because they share key features with normal stem cells (O'Brien et al., 2010). However, different classes of WNT signalling cascade inhibitors could be tested in cancer (Harb et al., 2019).

Overall, we have discussed hallmarks of amoeboid-specific cancer cell programs, and how these can be teased apart from their homeostatic functions within leukocyte populations. Additionally, it is important to define a window of opportunity for anti-amoeboid cancer therapy in which tumours have already been infiltrated. Moreover, treatment in the neo-adjuvant setting could be an option to prevent amoeboid cell-mediated recurrence. In conclusion, identifying targetable attributes of amoeboid cancer cells will be key to prevent cancer metastasis and therapy resistance.
